# Supplementary material for: Natural Root Cellular Variation in Responses to Osmotic Stress in Arabidopsis thaliana Accessions
Source: Genes (Basel). 2019 Nov 29;10(12):983. doi: 10.3390/genes10120983 (PMC6969899; doi:10.3390/genes10120983)
Supplement: Supplementary file 1 [file genes-10-00983-s001.pdf]

Natural root cellular variation in responses to osmotic stress in Arabidopsis *thaliana*

Wendy Cajero-Sanchez[+,1,2], Pamela Aceves-Garcia[+,1,2], María Fernández-Marcos[3], Crisanto Gutiérrez[3], Ulises Rosas[4], Berenice García-Ponce[1], Elena R. Álvarez-Buylla[1,5], Maria de la Paz Sánchez*[,1] and Adriana Garay-Arroyo*[,1,5].

Supplementary Material:

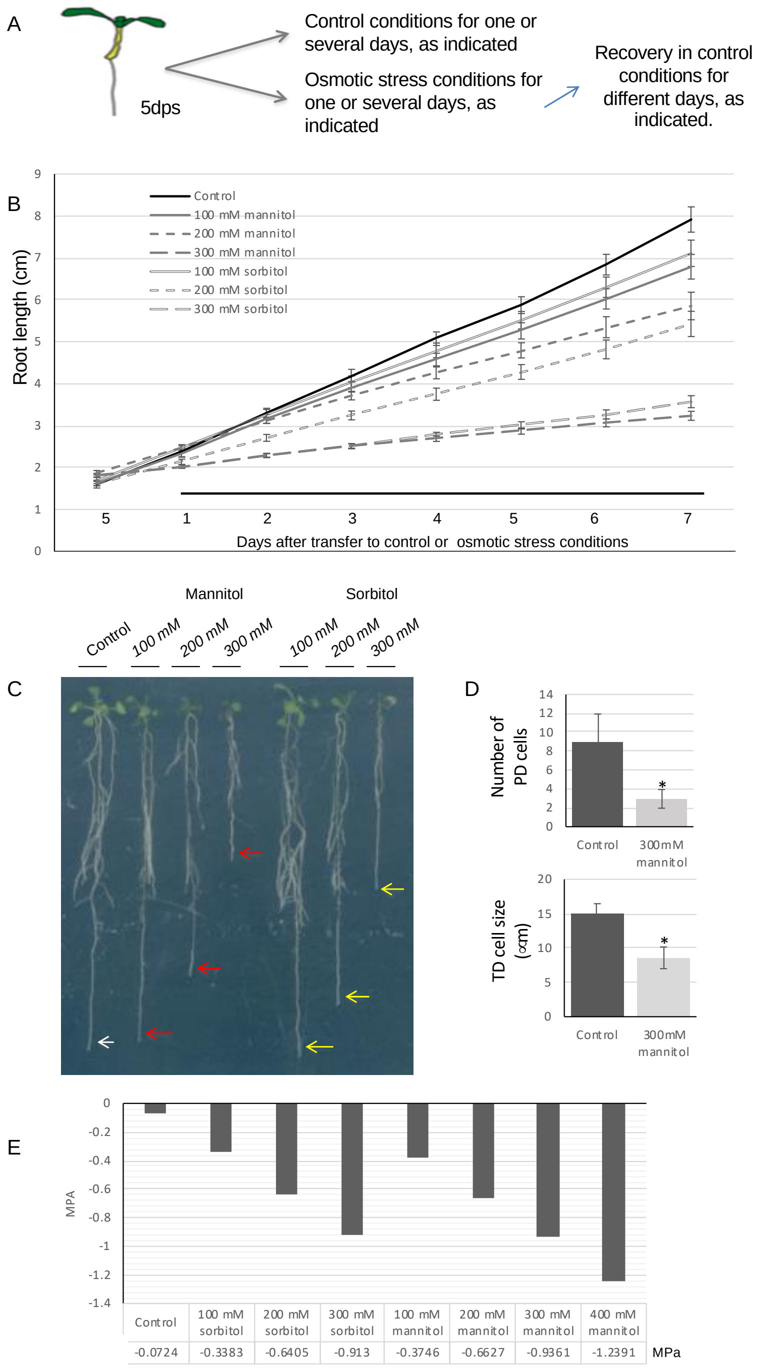

Supplementary Figure 1. Different osmotic stress conditions decrease root growth. (A) Cartoon showing the outline of the experimental procedure used in this work. (B) Growth curve of five-day old WT (Col-0) seedlings geminated on MS medium and transferred to control conditions or media supplemented with various concentrations of sorbitol or mannitol for the days indicated.

Values are means +/- SEM for 21 plants on 100 mM sorbitol, 17 plants on 200 mM sorbitol, 47 plants on 300 mM sorbitol, 26 plants on 100 mM mannitol, 23 plants on 200 mM mannitol and 50 plants on 300 mM mannitol. (C) Seedlings grown for 5 days under control conditions and then transferred to control or osmotic stress conditions; photographs were taken 8 days after transferred. (D) Cellular quantification of PD and TD root zones in control and 300mM Mannitol conditions. (E) Osmotic potential measures of control (MS 0.2X) and osmotic stress conditions of sorbitol (100, 200 and 300 mM) and mannitol (100, 200, 300 and 400 mM).

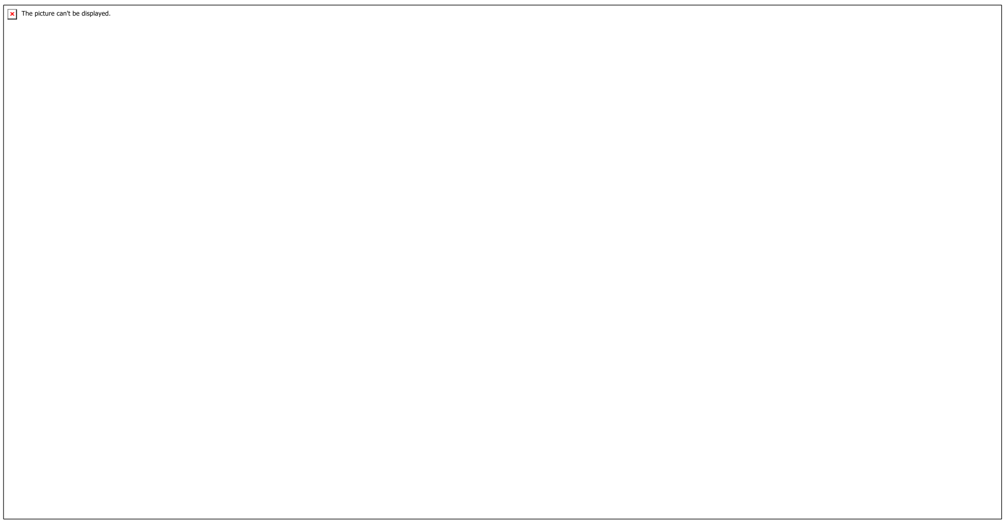

Supplementary Figure 2. Mannitol stress treatment does not affect the number of the TD cells neither the PD cell size but affects the elongation zone size. (A-C) Different cellular parameters that were measured under osmotic stress conditions

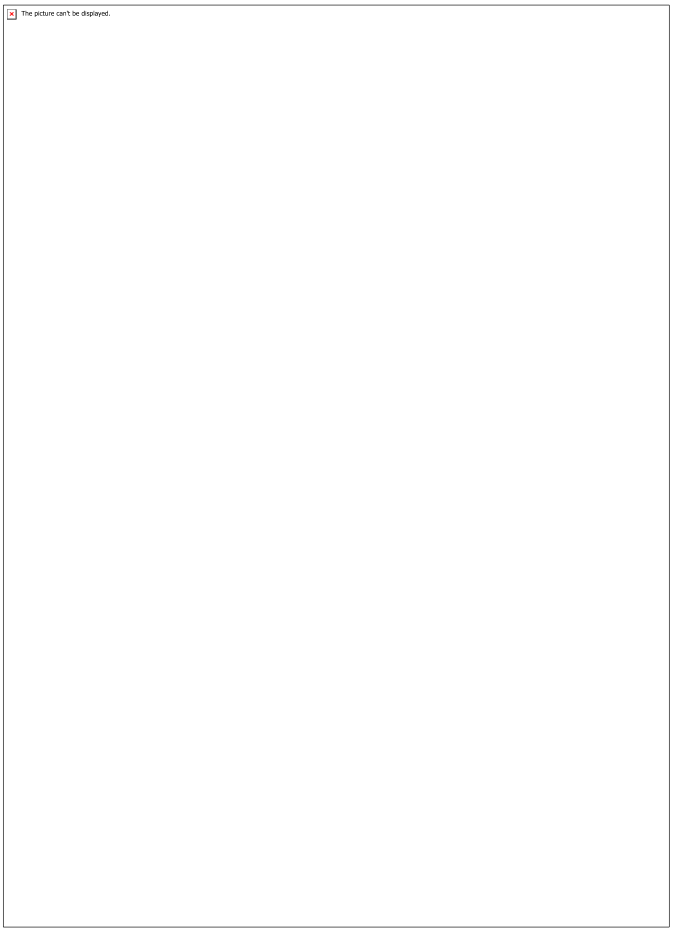

Supplementary Figure 3. Hyperosmotic stress conditions do not alter the QC or the SCN patterning of the root meristem. Confocal image of longitudinal optical sections of meristem roots at 5 d.p.s. for (A) Col-0 *WOX5:GFP* expression (100%; n=23) and (B) Col-0 *SCR:GFP* expression (100%; n=11) in control conditions and hyperosmotic stress conditions (300 mM mannitol); red signal is emitted by propidium iodide that was used as a counterstain. White arrow indicates the position of the QC (Bar = 25 µm).

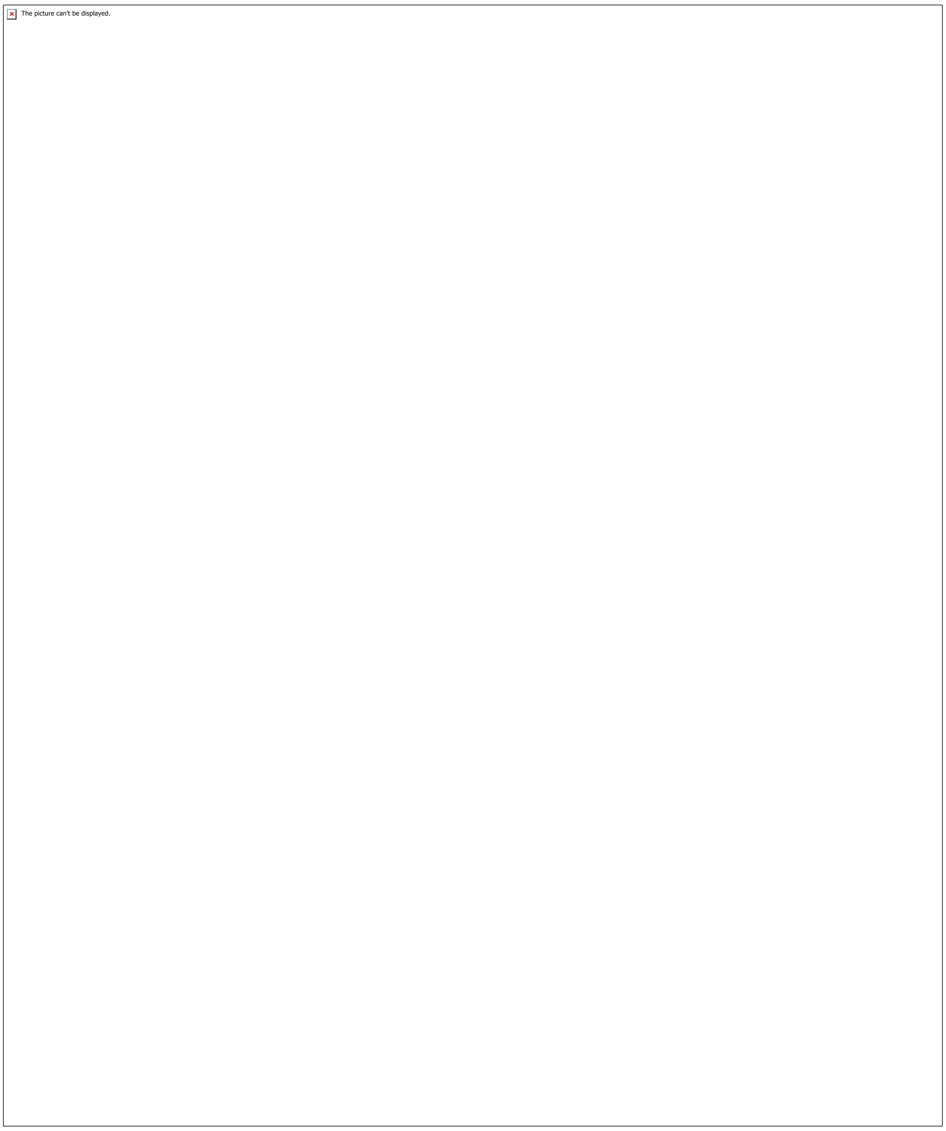

Supplementary Figure 4. Osmotic stress with different concentrations of sorbitol and mannitol cause radial enlargement of epidermal cells in the SZ. Median longitudinal confocal images of primary roots of plants grown for 5 days in MS and then transferred to control, 200 mM or 300 mM sorbitol and 200 mM mannitol for 1 day; n=12 in all cases. These plants also developed (as with 300 mM mannitol) short cells with hair roots in the SZ (see Figure 2).

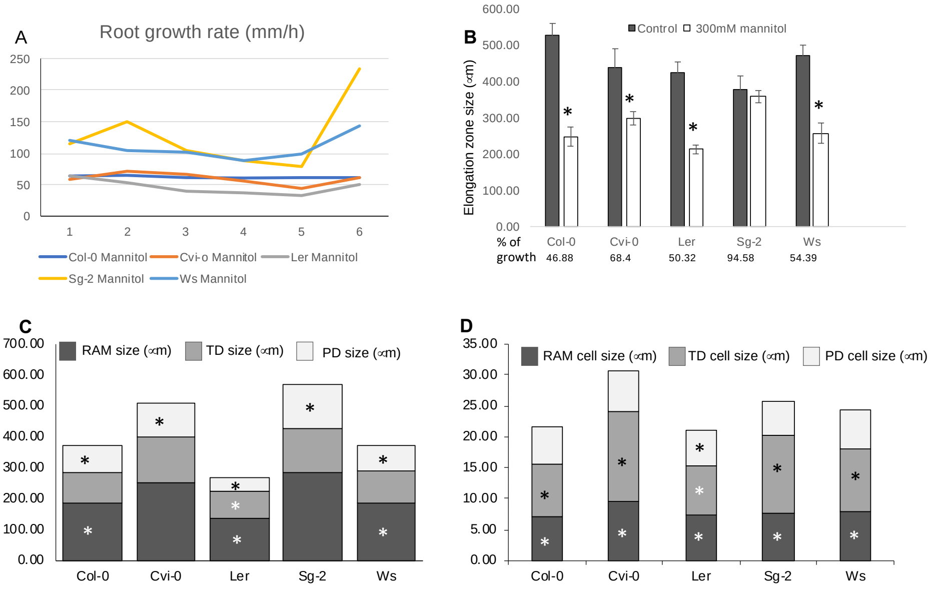

Supplementary Figure 5. (A) Effect of 300mM mannitol stress condition on root growth rate, the days of treatment were from 6-11. (B-D) Quantitative cellular parameters the five accessions in mannitol compared to their respective controls. Each of these parameters were obtained with the algorithm of Pacheco-Escobedo and collaborators (2016). Statistical analysis was carried out with a two-way ANOVA and a Tukey a post hoc analysis ($P \leq 0.05$).

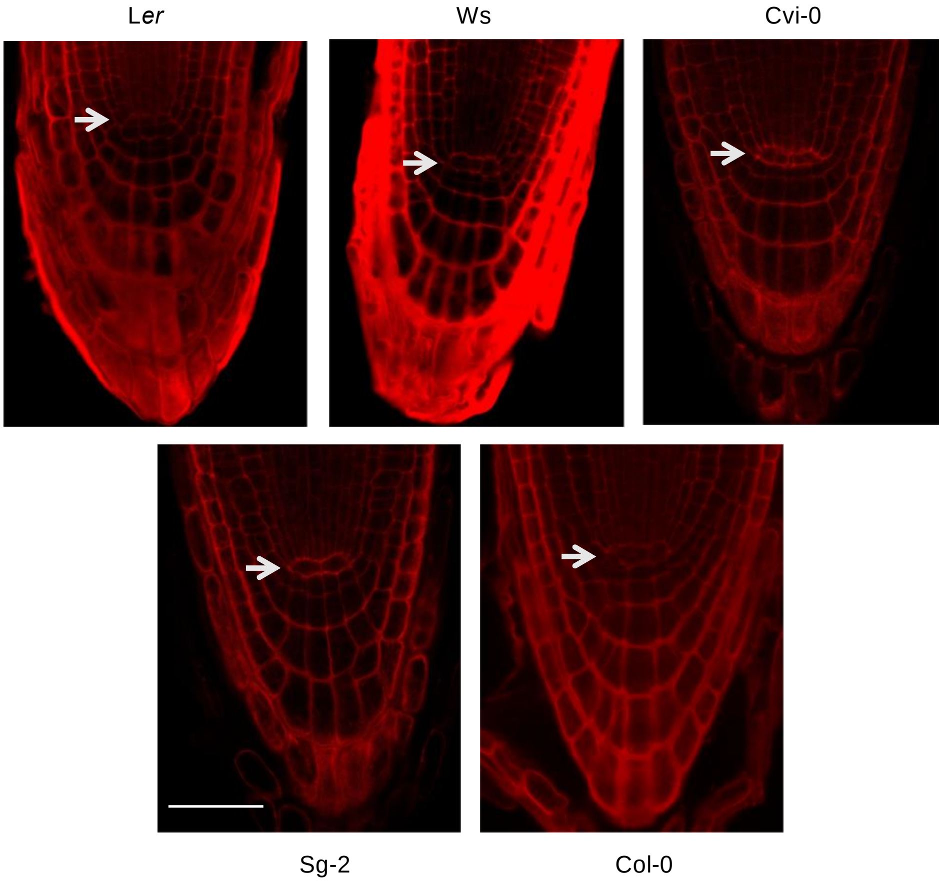

Supplementary Figure 6. Hyperosmotic stress conditions do not alter the QC nor the SCN patterning of the root meristem of the five accessions studied. Longitudinal confocal optical sections of the root SCN of five different accessions of Arabidopsis in osmotic stress conditions at 6 dps. The plants were grown 5 days in MS and then transferred 1 day to osmotic stress conditions. White arrow indicates the position of the QC (Bar = 50 µm).

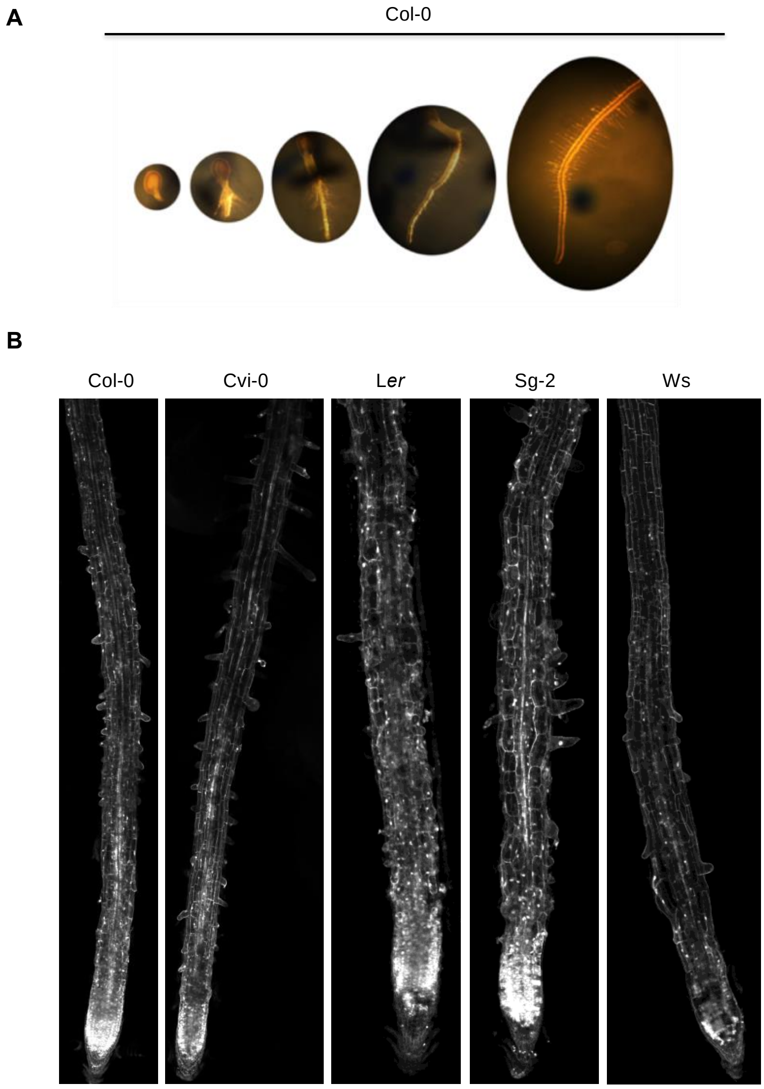

Supplementary Figure 7. Germination and plant growth under osmotic stress conditions (300 mM mannitol). (A) Col-0 seeds were germinated in mannitol 300 mM and kept under those stress conditions for several days; it is important to mention that, under this growth conditions, the plants do not generate a stress zone. (B) Median longitudinal confocal images of plants grown for ten days under hyperosmotic conditions (300 mM mannitol).

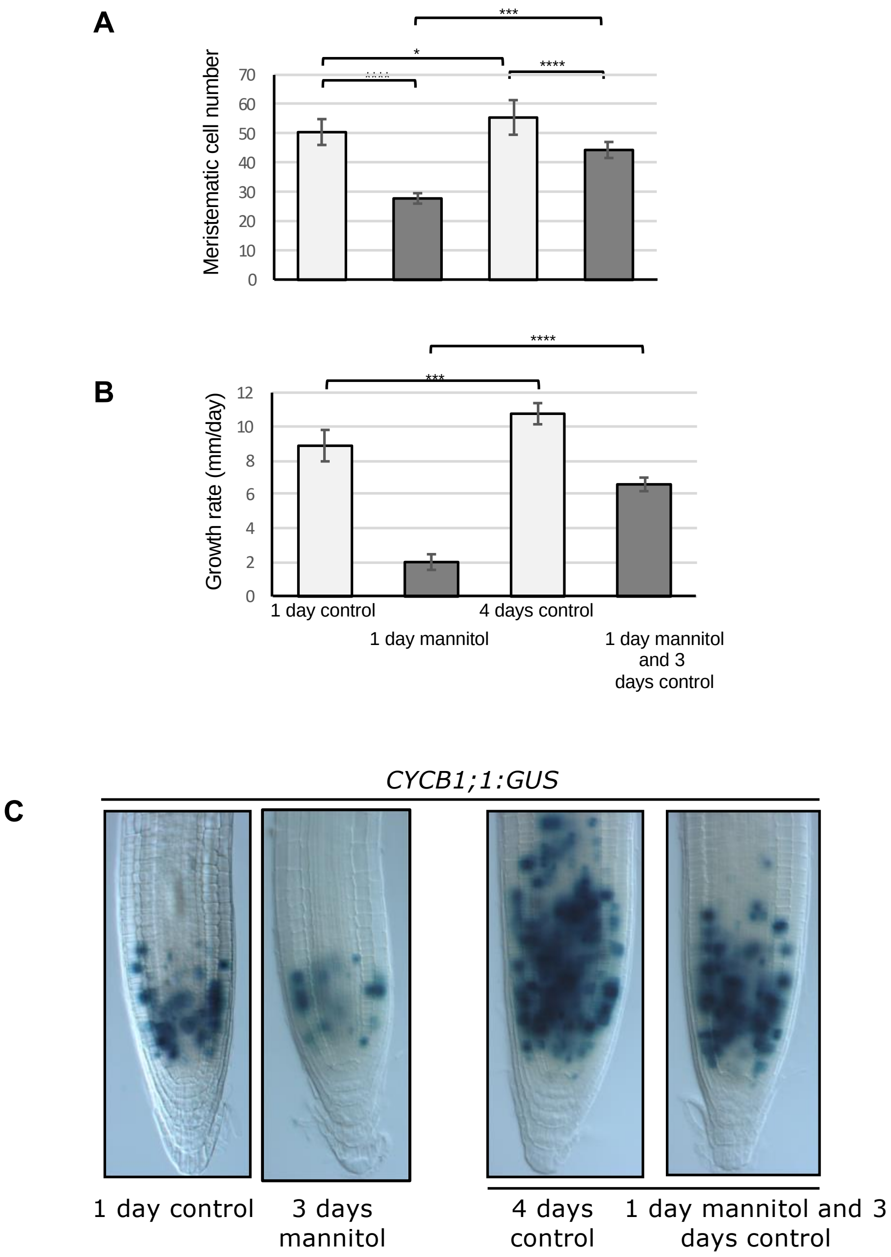

Supplementary Figure 8. Meristem number and growth rate of Col-0 after 1 day of hyperosmotic stress treatment and 3 days of recovery. (A) Meristem cell number of 6dps seedling growing for 5 day on control conditions and the transferred to control conditions or 300mM mannitol for one day. (B) Growth rate of 6dps seedling growing for 5 day on control conditions and the transferred to control conditions or 300mM mannitol for one day (1S) and one day on stress and three days of recovery. (C) Five-day-old *CYCB1;1DB-GUS* seedlings geminated on MS medium were transferred to control conditions or media supplemented with 300mM of mannitol for 1 day (n= 20) for 3 days (n= 32) for 1 day and then transferred to control conditions for 3 days as indicated (n= 28 control; n= 26 mannitol). **** P<0.0001; *** P<0.006; ** P< 0.002

Table 1. List of primers used for RT-qPCR assays

Gene	Primer	Sequence (5´-3´)
RD29A	FW	CTGATGAGGTGAAGCCAGAA
	RV	GAGCCAAGTGATTGTGGAGA
RD29B	FW	AAAGGAGCGGTCACTTCTTGG
	RV	AAACCCCATAGTCCCAACGGT
NCED3	FW	ACATGGAAATCGGAGTTACAG
	RV	GATGAATGTACCGTGAAATCC
COR15A	FW	AACGAGGCCACAAAGAAAGC
	RV	CCCAATGTATCTGCGGTTTC
RAB18	FW	CTCGGAGGATGATGGACAAG
	RV	TGAGCTTGACCAGACTGATC
PDF2	FW	TAACGTGGCCAAAATGATGC
	RV	GTTCTCCACAACCGCTTGGT
UPL7	FW	TTCAAATACTTGCAGCCAACCTT
	RV	CCCAAAGAGAGGTATCACAAGAGACT
